# Disparities in SARS-CoV-2 Vaccination-to-Infection Risk During the COVID-19 Pandemic in Massachusetts

**DOI:** 10.1001/jamahealthforum.2021.2666

**Published:** 2021-09-17

**Authors:** Scott Dryden-Peterson, Gustavo E. Velásquez, Thomas J. Stopka, Sonya Davey, Rajesh T. Gandhi, Shahin Lockman, Bisola O. Ojikutu

**Affiliations:** 1 Division of Infectious Diseases, Department of Medicine, Brigham and Women’s Hospital, Boston, Massachusetts; 2 Department of Immunology and Infectious Diseases, Harvard T.H. Chan School of Public Health, Boston, Massachusetts; 3 Botswana-Harvard AIDS Institute, Boston, Massachusetts; 4 Division of Global Health Equity, Department of Medicine, Brigham and Women’s Hospital, Boston, Massachusetts; 5 Department of Global Health and Social Medicine, Harvard Medical School, Boston, Massachusetts; 6 Department of Public Health and Community Medicine, Tufts University School of Medicine, Boston, Massachusetts; 7 Tufts Clinical and Translational Science Institute, Boston, Massachusetts; 8 Department of Urban and Environmental Policy and Planning, Graduate School of Arts and Sciences, Tufts University, Medford, Massachusetts; 9 Department of Community Health, School of Arts and Sciences, Tufts University, Medford, Massachusetts; 10 Department of Medicine, Brigham and Women’s Hospital, Boston, Massachusetts; 11 Division of Infectious Diseases, Massachusetts General Hospital, Boston

## Abstract

This cohort study examines the alignment of vaccination and SARS-CoV-2 risk in Massachusetts by creating and applying a vaccination-to-infection risk ratio.

## Introduction

Effective vaccine-based containment strategies for SARS-CoV-2 require equitable coverage of communities at greatest risk of infection.^1^ Adapting a concept from HIV-prevention efforts,^2^ we examined the alignment of vaccination and SARS-CoV-2 risk in Massachusetts by creating and applying a vaccination-to-infection risk (VIR) ratio.

## Methods

We aggregated community SARS-CoV-2 testing and vaccination data from the Massachusetts Department of Public Health and the Boston Public Health Commission from January 29, 2020, through June 24, 2021. Data were available for 293 distinct communities (278 cities and towns, and 15 Boston neighborhoods) with a cumulative population of 6 755 622 (98.6%). We adhered to Strengthening the Reporting of Observational Studies in Epidemiology (STROBE) reporting guidelines. This study involved the use of public anonymized data and was therefore designated as exempt by the Mass General Brigham Institutional Review Board.

We considered each community’s cumulative incidence of confirmed SARS-CoV-2 infections to be the best available indicator of future infection risk. We used 2 approaches to assess vaccination equity: VIR ratio and Lorenz curves. The VIR ratio was calculated for each community as the quotient of the number of fully vaccinated individuals divided by the cumulative number of confirmed SARS-CoV-2 infections. Communities with VIR ratios below the statewide mean have lower vaccination coverage relative to their infection risk. Lorenz curves, which assess equity in resource distribution,^3^ were used to describe vaccination relative to COVID-19 burden and calculate summaries of inequity (Gini index) and magnitude of vaccine reallocation required to achieve equity (Hoover index).

We used population estimates from the Massachusetts Department of Public Health and the American Community Survey to determine community age, race, and ethnic composition. Socioeconomic vulnerability was estimated using the Socioeconomic Status domain of the Social Vulnerability Index (census tract ranks aggregated by community). We fit a negative binomial model using robust sandwich estimators to assess associations between community VIR ratios and a priori–selected predictors: proportion 65 years or older (an early vaccination eligibility criterion), proportion identified as Black and/or Latinx individuals (<20% or ≥20%), quartile of socioeconomic vulnerability, and community size (<7500 or ≥7500 residents). Analyses were conducted in R, version 4.0.5 (R Foundation), and a 2-tailed *P* < .05 was considered statistically significant.

## Results

**Figure 1.**
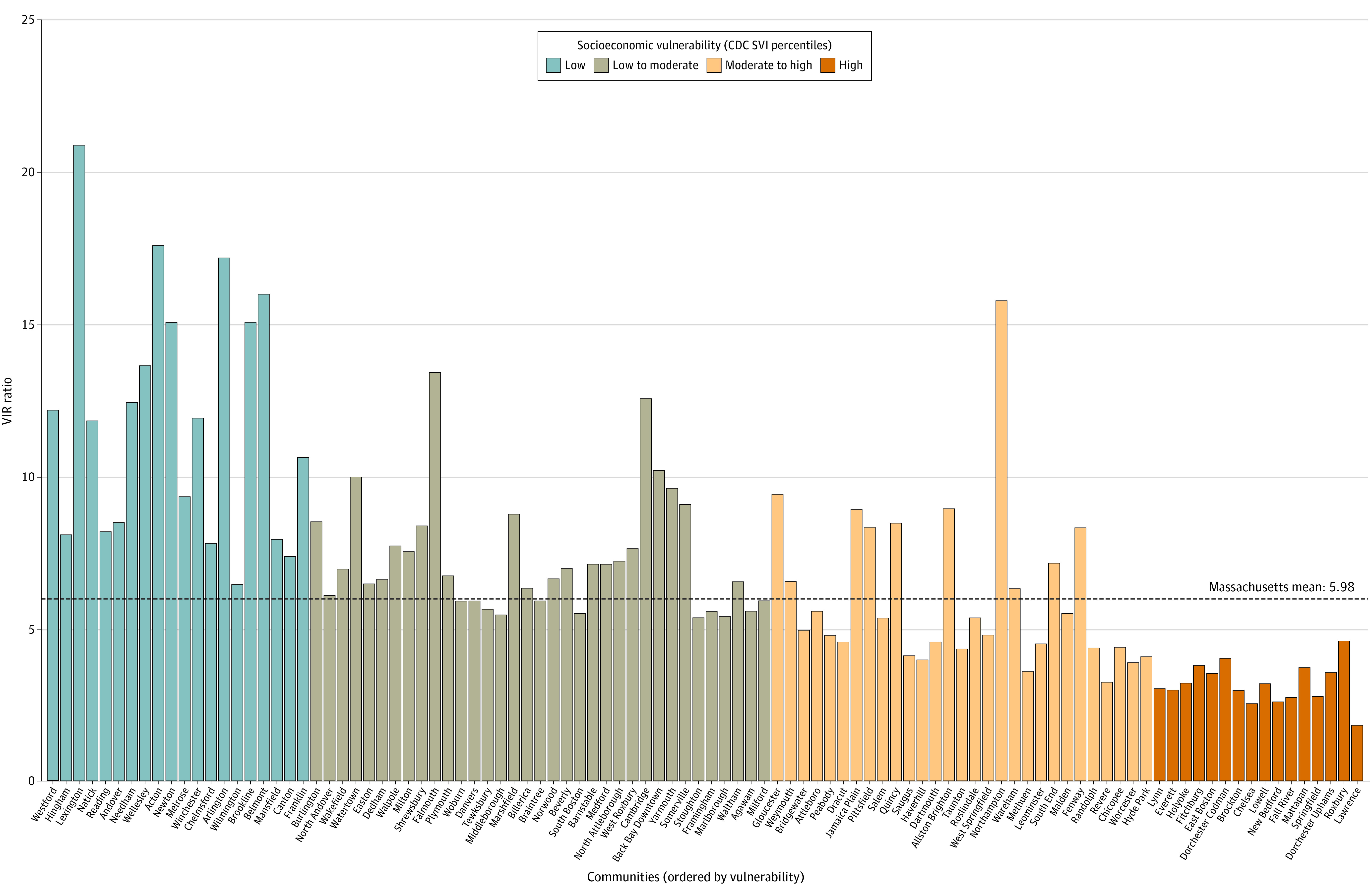
Vaccination-to-Infection Risk (VIR) Ratio Among the 100 Largest Massachusetts Communities, Ordered by Socioeconomic Vulnerability The VIR ratio is calculated as the cumulative number of fully vaccinated individuals divided by the cumulative number of confirmed SARS-CoV-2 infections in each community reported from January 29, 2020, through June 24, 2021. Mean VIR by socioeconomic quartile: low,10.8; low to moderate, 7.36; moderate to high, 5.40; and high, 2.99. Socioeconomic vulnerability was estimated using the Socioeconomic Status domain of the Centers for Disease Control and Prevention (CDC) Social Vulnerability Index (SVI).

As of June 24, 2021, 649 379 (9.6%) SARS-CoV-2 infections had been confirmed in 6 755 622 residents of included communities, and 3 880 706 (57.4%) were fully vaccinated. Cumulative incidence of confirmed SARS-CoV-2 infection (minimum, 1.6%; maximum, 24.1%) and complete vaccination (minimum, 26.5%; maximum, 99.6%) varied considerably between communities. Communities with increased socioeconomic vulnerability had lower VIR ratios indicating less equitable vaccination relative to infection risk (Figure 1).

In multivariable analysis, decreased vaccination relative to infection risk was independently associated with increasing socioeconomic vulnerability (adjusted relative risk [aRR], 0.82 per quartile increase; 95% CI, 0.76-0.87; *P* < .001) and when more than 20% of the community identified as Black and/or Latinx individuals (aRR, 0.73; 95% CI, 0.62-0.86; *P* < .001). Improved community vaccine coverage was associated with higher community proportion of residents 65 years or older (aRR, 1.11 per 5% increase in proportion; 95% CI, 1.03-1.20; *P* = .007) and community size fewer than 7500 residents (aRR, 1.48; 95% CI, 1.30-1.68; *P* < .001).

**Figure 2.**
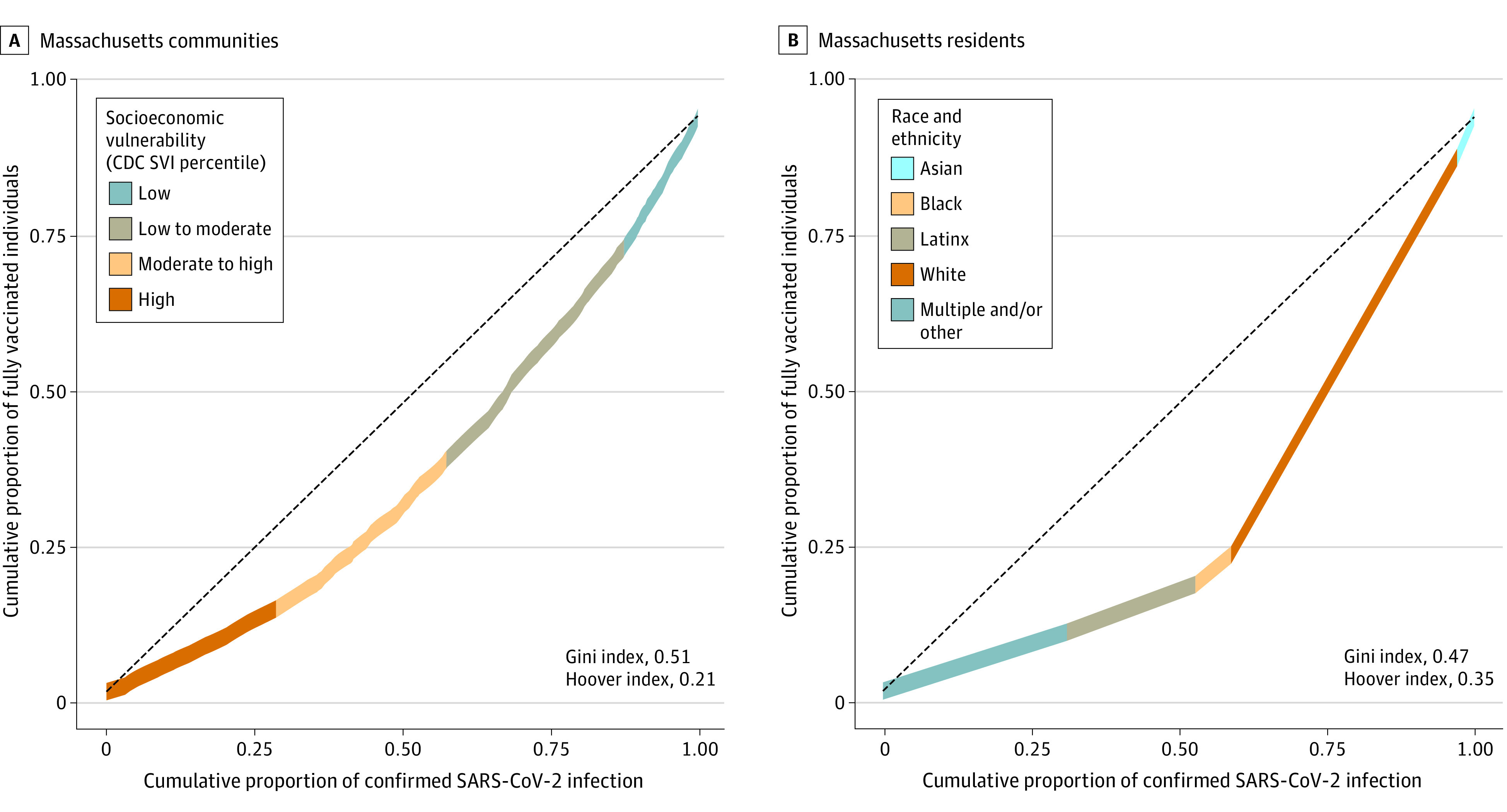
Lorenz Curves of Cumulative Proportion of Vaccination and COVID-19 Infection by Community and Individual Residents in Massachusetts Asian, Black, and Latinx residents, as well as residents of multiple and/or other races and ethnicities (including American Indian and Alaska Native, Native Hawaiian and Pacific Islander, and other and unspecified/unknown races) accounted for 62% of confirmed infections and 30% of full vaccinations. Cases and vaccinations reported from January 29, 2020, through June 24, 2021, are included. Socioeconomic vulnerability was estimated using the Socioeconomic Status domain of the Centers for Disease Control and Prevention (CDC) Social Vulnerability Index (SVI).

Lorenz curves indicated considerable inequity, with an estimated Gini coefficient (1, complete equity; 0, complete inequity) of 0.51 between communities and 0.47 by race and ethnicity of individuals (Figure 2). An estimated 810 000 full vaccination courses would need to be diverted to undervaccinated communities to achieve equity.

## Discussion

In this cohort study, analysis of SARS-CoV-2 vaccination indicated structural disparity in vaccine distribution with lower vaccine coverage to infection risk in communities with increased socioeconomic vulnerability and larger proportions of Black and Latinx individuals. While a limitation of this study is that these analyses do not directly assess the mechanisms of disparity, Massachusetts prioritized large hospital systems and mass vaccination sites^4^ rather than strategies to mitigate structural racism recommended by others.^5,6^ In conclusion, disparities in vaccine coverage highlight ongoing inequities in the approach to COVID-19 and imperil efforts to control the pandemic.

## References

[1] Wrigley-Field E, Kiang MV, Riley AR, . Geographically-targeted COVID-19 vaccination is more equitable than age-based thresholds alone. medRxiv. Preprint posted online March 27, 2021. doi:10.1101/2021.03.25.21254272 PMC8480919 34586843

[2] Siegler AJ, Mouhanna F, Giler RM, . The prevalence of pre-exposure prophylaxis use and the pre-exposure prophylaxis-to-need ratio in the fourth quarter of 2017, United States. Ann Epidemiol. 2018;28(12):841-849. doi:10.1016/j.annepidem.2018.06.005 29983236 PMC6286209

[3] Mody A, Pfeifauf K, Geng EH. Using Lorenz curves to measure racial inequities in COVID-19 testing. JAMA Netw Open. 2021;4(1):e2032696. doi:10.1001/jamanetworkopen.2020.32696 33416882 PMC7794664

[4] Goralnick E, Kaufmann C, Gawande AA. Mass-vaccination sites—an essential innovation to curb the Covid-19 pandemic. N Engl J Med. 2021;384(18):e67. doi:10.1056/NEJMp2102535 33691058

[5] Bibbins-Domingo K, Petersen M, Havlir D. Taking vaccine to where the virus is—equity and effectiveness in coronavirus vaccinations. JAMA Health Forum. 2021;2(2):e210213. doi:10.1001/jamahealthforum.2021.0213 36218794

[6] Corbie-Smith G. Vaccine hesitancy is a scapegoat for structural racism. JAMA Health Forum. 2021;2(3):e210434. doi:10.1001/jamahealthforum.2021.0434 36218456

