# Error in Results and Additional Information Section Added to End Matter

**DOI:** 10.1001/jamahealthforum.2021.3871

**Published:** 2021-11-05

The Research Letter titled, “Disparities in SARS-CoV-2 Vaccination-to-Infection Risk During the COVID-19 Pandemic in Massachusetts,”^1^ published online September 17, 2021, included a data error in the Results section. The percentage of 649 379 SARS-CoV-2 infections among 6 755 622 residents of included communities in the study is 9.6%, rather than 8.9%. An Additional Information section was also added to the end matter. The article has been corrected online.
